# Lessons learnt from the Bristol Girls Dance Project cluster RCT: implications for designing and implementing after-school physical activity interventions

**DOI:** 10.1136/bmjopen-2015-010036

**Published:** 2016-01-08

**Authors:** Mark J Edwards, Thomas May, Joanna M Kesten, Kate Banfield, Emma L Bird, Jane E Powell, Simon J Sebire, Russell Jago

**Affiliations:** 1 Centre for Exercise Nutrition & Health Sciences, School for Policy Studies, University of Bristol, Bristol, UK; 2 Health and Social Sciences, University of the West of England, Bristol, UK; 3 School of Social and Community Medicine, University of Bristol, Bristol, UK

**Keywords:** QUALITATIVE RESEARCH, PREVENTIVE MEDICINE, PUBLIC HEALTH, PHYSICAL ACTIVITY, PROCESS EVALUATION

## Abstract

**Objective:**

To consider implementation issues associated with the delivery of Bristol Girls Dance Project (BGDP) and to identify improvements that may aid the design of after-school physical activity (PA) interventions.

**Design:**

Two-armed cluster randomised control trial. The BGDP was a 20-week school-based intervention, consisting of two 75 min after-school dance sessions per week, which aimed to support Year 7 girls to be more physically active.

**Setting:**

18 secondary schools (nine intervention, nine control) in the Greater Bristol area (as an indication of deprivation, children eligible for the pupil premium in participant schools ranged from 6.9 to 53.3%).

**Participants:**

571 Year 7 girls. This article reports on qualitative data collected from 59 girls in the intervention arm of the trial, 10 dance instructors and 9 school contacts involved in the delivering of the BGDP.

**Methods:**

Data were obtained from nine focus groups with girls (one per intervention school), and interviews with dance instructors and school contacts. Focus groups sought views of girls’ motivation to participate, teaching styles and experiences of the intervention. Interviews explored views on implementation and dissemination. Framework analysis was used to analyse data.

**Results:**

Qualitative data elicited three themes associated with the delivery of BGDP that affected implementation: project design, session content and project organisation. ‘Project design’ found issues associated with recruitment, timetabling and session quantity to influence the effectiveness of BGDP. ‘Session content’ found that dance instructors delivered a range of content and that girls enjoyed a variety of dance. Themes within ‘project organisation’ suggested an ‘open enrolment’ policy and greater parental involvement may facilitate better attendance.

**Conclusions:**

After-school PA interventions have potential for increasing PA levels among adolescent girls. There is a need to consider the context in which interventions are delivered and implement them in ways that are appropriate to the needs of participants.

**Trial registration number:**

ISRCTN52882523.

**Strengths and limitations of this study**

- Relevance beyond after-school dance interventions for researchers and practitioners designing and delivering after-school interventions.
- Study focuses on the significance of the context in which the intervention is delivered.
- Data obtained from in-depth qualitative interviews with participants and key stakeholders.
- Large sample of participants (n=78) for the qualitative study and evidence of data saturation.
- Trial methodology limits generalisations.

## Introduction

Ensuring that all members of society are physically active is important for public health. Physical activity (PA) is associated with improved physical and mental well-being among children and young people.^1–3^ A number of studies have shown that large proportions of young people do not engage in the recommended hour of moderate-to-vigorous PA (MVPA) per day.^4^ ^5^ Girls are often found to be less active than boys across childhood and adolescence and, as such, there is a need for interventions to encourage more PA in girls, particularly during the transition into adolescence when the decline in female PA is at its highest.^6–8^ Girls tend to be more sedentary and also engage in less MVPA than boys.^9^ A study examining barriers faced by girls to PA suggests that safety concerns, the competitive nature of many activities, inaccessible facilities and body-image concerns are key perceived barriers to girls being active.^10^ Additionally, girls face more restrictions than boys in terms of their freedom to play outdoors.^8^ Dance is an activity that could resolve a number of these barriers and as such it is popular among adolescent girls in the UK, and could therefore be an appropriate activity to increase girls’ PA.^4^ ^11–13^

Schools are a good place to target interventions as attendance is a legal requirement. PA interventions delivered during the school-day have had limited effect,^7^ ^8^ ^12^ ^14^ suggesting a need to consider alternative school-based interventions.^4^ ^14^ ^15^ Pate and O'Neill^14^ suggest that the quest for academic excellence combined with resource limitations restricts opportunities for PA within the school day. Several systematic reviews have highlighted the potential of extracurricular PA interventions for young people, however, there is a lack of robust evaluations of these programmes.^7^ ^12^ Incorporating dance into after-school activities could contribute to overall PA among girls failing to achieve the recommended UK PA guidelines.^4^ ^11^ As such, the Bristol Girls Dance Project (BGDP) examined the potential of an after-school dance-based intervention targeted at increasing PA levels of Year 7 (age 11–12) girls.

A feasibility trial was conducted to assess the potential of a dance-based intervention.^4^ This formative work found that it was possible to recruit adolescent girls to an after-school dance intervention and that such an intervention could yield positive effects on their PA. The process evaluation reported fluctuating attendance and low perceived exertion levels within sessions. Additionally, post-intervention qualitative work suggested that a reduction in the time allocated for ‘creative’ tasks, better behaviour management guidance, and exposure to a wider range of dance styles would improve the intervention.^4^ The intervention was refined in light of these findings and tested in a fully powered cluster randomised controlled trial,^16^ on which the present paper reports.

BGDP was a 20-week school-based two-armed cluster randomised control trial. The intervention consisted of two 75 min after-school dance sessions per week for Year 7 (11–12 years) girls in the intervention arm. Intervention sessions were delivered by professional dance instructors who attended training led by study staff. The training introduced instructors to the study aims and rationale, the BGDP intervention sessions and the underpinning Self-Determination Theory (SDT).^17^ ^18^ Session plans underpinning the BGDP sessions encouraged dance instructors to use a variety of dance styles throughout the course of the intervention (encouraging participant choice in this was strongly encouraged).

The BGDP aimed to increase autonomous motivation for dance and PA among participants. The dance instructor training and BGDP session plan manual were integral to this aim. The SDT-focused element of the training explored the practical application of the theory to dance sessions. Instructors were provided the opportunity to use autonomy-supportive styles of instruction, seek clarification and obtain feedback from study staff. Behaviour management was discussed and further details included in the session plan manual. Halfway through the intervention period the instructors attended a half-day booster session that recapped study aims, the application of SDT in sessions, and provided a forum to discuss issues that arose during session delivery.

Full details of the trial protocol^13^ and results have been published elsewhere (R Jago, M J Edwards, S J Sebire, *et al*. Effect and cost of an after-school dance programme on the physical activity of 11–12year old girls: the Bristol girls dance project school-based cluster randomised controlled trial. *Int J Behav Nutr Phys* Under review). Briefly however, there was no difference in PA levels between the intervention and control group girls during the last few weeks of the intervention or at 6-month follow-up. Findings reported elsewhere showed that intervention fidelity was generally good, with high levels of enjoyment among participants (S J Sebire, M J Edwards, J M Keston, *et al*. Process evaluation of the Bristol girls dance project. *BMC Public Health* Under review). However, session attendance was highly variable with only one-third of girls attending two-thirds of the sessions. Attendance also declined during the project.

Process evaluations are central to understanding how complex interventions work^19^ by focusing on the processes of intervention delivery, receipt and fidelity.^19^ ^20^ When they are too narrowly focused, however, they can neglect to evaluate the broader contextual factors associated with individual agency, and the social context in which an intervention is delivered.^21^ It is important to understand how logistical arrangements, operations and implementation of intervention components contribute to intervention processes, and to also acknowledge the influence of dance instructors delivering the intervention in a specific context. Thus, there is a need to identify factors that enable effective intervention delivery and establish how these factors can be influenced. The aim of this paper is to use qualitative process evaluation data to document the lessons learnt from the BGDP and to identify key points for improvement that may increase attendance rates and improve overall delivery of future after-school school-based PA interventions.

## Methods

Eighteen schools participated in the study. All schools were located within 25 miles of Bristol city centre, and fell under the Bristol City, Bath and North East Somerset, or North Somerset Council areas. Schools were urban and suburban and in terms of deprivation they were slightly less deprived than the national average. Between 6.9% and 53.3% (average=26.2%) of pupils in study schools were eligible for the ‘pupil premium’, a form of governmental funding aimed at increasing the attainment of disadvantaged pupils (higher percentage equals greater deprivation).^22^ The national average is 27.8% of secondary pupils. Four of the nine intervention schools had above average levels of deprivation.

All Year 7 girls eligible to take part in physical education were invited to participate (n=1877). There was space for 33 girls to take part in each school. Recruitment consisted of a ‘taster’ session that provided exposure to a typical intervention session, a briefing, and written information for girls and parents/guardians. Six hundred and thirty three girls returned parental consent forms, of which 571 were selected at random (due to the maximum limit of 33 girls per school). Participants completed four sets of measurements (accelerometer, psychosocial questionnaire and height and weight) at three time-points (baseline, T1 (end of intervention period), and T2 (baseline+52 weeks)). Girls received a £10 thank you voucher for completing each measurement stage. Schools were randomised to control (n=9) or intervention (n=9) arm after baseline measures, with 284 girls in the intervention and 287 in the control arm.

The present study draws on interview data collected soon after the intervention ended from dance instructors (n=10) who delivered the intervention and school contacts (n=9) who facilitated intervention logistics in their school. School contacts were the study team's main point of contact with the school. These individuals were four physical education (PE) staff, one Year 7 teacher, three dance teachers and one drama teacher. Nine focus groups were conducted with girls that received the intervention (n=59, range=3–8). Ten girls from each intervention school, reflecting different tertiles of attendance, were invited. This was in order to capture a range of participant views. Girls who attended ≤3 sessions were not included as they would be unable to answer a significant proportion of the topic guide questions. Further details of participant sampling, recruitment and reasons for why children stopped attending intervention sessions are reported elsewhere (S J Sebire, *et al*. Under review). For dance instructors, interviews explored views on the implementation and dissemination of BGDP. School contact interviews focused on how the intervention was delivered and areas for improvement. Focus groups among girls explored motivations to participate, dance instructor teaching style, and experiences of the intervention. Interview guides are included as online supplementary files 1–3 for participant focus groups, dance instructors and school contact interviews, respectively. School contact interviews and participant focus groups were conducted in schools and dance instructor interviews were conducted in convenient locations for participants (eg, cafes). All interviews and focus groups were audio recorded and transcribed verbatim. Transcripts were compared with the recordings and amended as necessary.

Ethical approval was obtained from the School for Policy Studies ethics and research committee at the University of Bristol. Written parental consent was obtained for all children who participated in the study and informed consent was gained from the dance instructors and school contacts who participated. A CONSORT extension for Cluster Trials Checklist has been completed.

### Analysis

A framework analysis was used.^23^ The framework method is a seven stage procedure for analysing qualitative data, characterised by detailed line-by-line coding and the charting of data into a framework matrix.^23^ Initial codes were created openly using NVivo (V.10, QSR International) to categorise transcripts into components that were of potential significance to the research objective. Codes were produced independently by four qualitative researchers (JMK, MJE, SJS and TM) who coded three transcripts each (one dance instructor, school contact and participant focus group). Initial codes formed a coding framework which was applied to the remaining transcripts. A predefined ‘school context’ code was included to identify differences in delivery between schools. Frameworks were subsequently triangulated to substantiate the relationships between all three informant groups. The qualitative research team met weekly to discuss and iteratively refine the codes, which led to the production of the three coding frameworks (one for each respondent group). Illustrative quotes capturing the essence of each theme were identified and agreed by the researchers. A COREQ checklist for reporting of qualitative studies is included (table 1).

**Table 1:** Consolidated criteria for reporting qualitative studies (COREQ): 32-item checklist

No	Item	Guide questions/description
*Domain 1: Research team and reflexivity*		
Personal characteristics		
1.	Interviewer/facilitator	Which author/s conducted the interview or focus group? **JK, ME**
2.	Credentials	What were the researcher's credentials? *For example, PhD, MD* **PhD**
3.	Occupation	What was their occupation at the time of the study? **Research Associate**
4.	Gender	Was the researcher male or female? **Female (JK); Male (ME)**
5.	Experience and training	What experience or training did the researcher have? **Coverage of qualitative methodology and interview technique in PhD. Formal training on qualitative research methods from at BSc/BA and MSc.**
Relationship with participants		
6.	Relationship established	Was a relationship established prior to study commencement? **No**
7.	Participant knowledge of the interviewer	What did the participants know about the researcher? for example, *personal goals, reasons for doing the research* **Both JK and ME had met the interviewees on several occasions. ME recruited them to the study and JK conducted process evaluation while they were delivering the intervention.**
8.	Interviewer characteristics	What characteristics were reported about the interviewer/facilitator? for example, *Bias, assumptions, reasons and interests in the research topic* **None**
*Domain 2: study design*		
Theoretical framework		
9.	Methodological orientation and Theory	What methodological orientation was stated to underpin the study? for example, *grounded theory, discourse analysis, ethnography, phenomenology, content analysis* **Study was underpinned by self-determination theory. Qualitative analysis was conducted using a framework analysis**
Participant selection		
10.	Sampling	How were participants selected? for example, *purposive, convenience, consecutive, snowball* **Purposive sampling for qualitative focus groups. All dance instructors delivering the intervention and all school contacts were interviewed/**
11.	Method of approach	How were participants approached? for example, *face-to-face, telephone, mail, email* **Focus groups were conducted face to face** **Interviews with dance instructors conducted face to face** **One interview with a school contact was conducted via telephone. The remaining interviews were conducted face to face.**
12.	Sample size	How many participants were in the study? **Semi-structured interviews were conducted with all dance instructors who delivered the intervention (n=10) and school contacts (n=9) in intervention schools. A focus group (n=9) was conducted with girls who participated in each intervention school (n=59).**
13.	Non-participation	How many people refused to participate or dropped out? Reasons? Twelve participants withdrew from the study. 6 no longer wanted to participate 4 had illness(es) 1 relocated 1 excluded from school
Setting		
14.	Setting of data collection	Where was the data collected? for example, *home, clinic, workplace* **All focus groups conducted in schools. One school contact interview conducted via phone, all remaining conducted in school. Dance instructor interviews conducted in a range of settings.**
15.	Presence of non-participants	Was anyone else present besides the participants and researchers? **No**
16.	Description of sample	What are the important characteristics of the sample? for example, *demographic data, date* Focus group: All Year 7 girls. Dance instructor interviews: All female School contacts: All teaching staff. One male, the remaining female.
Data collection		
17.	Interview guide	Were questions, prompts, guides provided by the authors? Was it pilot tested? **Yes. No pilot conducted with final version of interview guide.**
18.	Repeat interviews	Were repeat interviews carried out? If yes, how many? **No**
19.	Audio/visual recording	Did the research use audio or visual recording to collect the data? **Audio recordings made for each interview/focus group.**
20.	Field notes	Were field notes made during and/or after the interview or focus group? **No.**
21.	Duration	What was the duration of the interviews or focus group? Average length Focus group: average length=42.38 min (range=30.35–50.23 min) Dance instructor interviews: average length=67.20 min (range=41.35–91.36 min) School contact interviews: average length=29.35 min (range=22.07–38.41 min)
22.	Data saturation	Was data saturation discussed? **Yes**
23.	Transcripts returned	Were transcripts returned to participants for comment and/or correction? **No**

We aimed to address issues that could be edited to improve future roll-out of similar interventions. Specifically, the issues addressed in this paper are:

- Why participants (school teachers, girls and dance instructors) took part in the study
- The acceptability of the design and content of the dance sessions
- Feedback on the intervention structure (eg, session quantity and duration)
- Views on the organisation of the study.

## Results

Three main themes associated with BGDP delivery were identified in the qualitative analysis. These related to: (1) project design; (2) session content; and (3) project organisation. The findings are presented by theme, and the subthemes include illustrative quotes from the different participant groups.

### Project design

Project design encompasses subthemes concerning BGDP logistical arrangements, including participant recruitment, timetabling, session quantity and project duration.

#### Recruitment

Different methods of recruitment were required for each participant group (ie, girls, dance instructors and school contacts).

##### School contacts

No expectations or requirements were expressed by the study team regarding what school contacts would need to do for the study, beyond a general breakdown of what the school's participation entails. Similarly, no school contact sought detailed instruction on what their role would necessitate. School contacts cited various reasons for their involvement in the project, with some describing a personal interest and others being asked by a colleague to act as a key contact.

I was asked by the Head of Year 7 because he had too much on his plate.

School contact 21

I think it was just sent generally to the school like a pack…there was quite a lot of information there so I just emailed ‘em through.

School contact 72

Two school contacts embraced a type of ‘research altruism’. One noted how their own degree meant they were familiar with research and were keen to engage with a research project:

I also liked that it was part of a research project as well. I've been doing a university degree myself and dissertations and […] it's really important that these things are done to try and take things forward.

School contact 23

##### Dance instructors

Dance instructor involvement in the project was motivated by numerous reasons. The research aspect of the project appealed to some instructors who viewed the project as an opportunity to disseminate their view of dance as a positive activity for young people:

I love to dance and I love to teach dance and to share my passion with as many people as possible. So any opportunity I'm interested in. I was really attracted to the project as a whole, the research that was involved.

Dance instructor 61

Dance instructors also viewed their involvement as an opportunity to develop teaching experience via the delivery of new dance styles:

The fact that we were delivering different styles of dance that was also really good for me because I haven't really done much else in terms of teaching, so it kind of pushed me to try different things which I did and then gained more confidence so I've gained more skills.

 Dance instructor 61

##### Girls

For some girls, the opportunity to try a new activity and learn new dance styles motivated participation:

I kind of just decided myself because I wanted to go like start something that I hadn't done before.

Focus group 23

I'm not a fan of dance but because I wanted to try something new so I tried it.

Focus group 62

For some girls involvement was based on spending time with their peers:

I was looking at some [afterschool clubs] but I was only really going to do them if like someone, like a friend, did it with me.

Because I didn't really want to go on my own and everyone else knew each other and I just turned up.

Focus group 61

Girls were given a £10 gift voucher for completing each phase of data collection. In two schools gift vouchers were interpreted as incentives to attend dance sessions by some. Indeed, one girl noted that participants should not receive a voucher unless they attend dance sessions.

You get a voucher. People signed up because of that. But I don't think they really signed up because they wanted to do the dance.

Focus group 53

In one focus group, being part of BGDP was experienced as a privilege because others were denied the opportunity (due to the limit of 33 girls per school):

It was like a privilege to like get into it because quite a lot of people like wanted to join but only a few of us did.

School contact 32

#### Timetabling

Some schools arranged BGDP sessions at a similar time to other after-school clubs, this led to different clubs/activities competing for attendance. However, in some schools, the time between the end of the school day and the beginning of BGDP sessions was short, meaning participants struggled to arrive punctually. This resulted in some sessions being short:

Partly it is to do with the set up at the school […] it's just a very annoying system that's in this school that because of the meetings that take place on a Tuesday and a Wednesday and we finish early on a Friday, Monday and Thursday are the only times available for any after school clubs. So all of the after school clubs run on a Monday and a Thursday. So you're all vying for kids.

School contact 62

After school finished we started five minutes later. That was not enough time. They needed ten minutes.

Dance instructor 51

#### Session quantity and project duration

School contacts suggested that the quantity of sessions (n=40) was too high to sustain attendance over the course of 20 weeks. Two sessions per week was also seen as a burden for girls by school contacts, especially when competing against other sporting events and social commitments:

I just feel that two sessions per week, and the length of time that it runs for, is possibly a bit too much to keep the attendance up.

School contact 72

I think possibly because it was so… on for such a long time they found it really hard to maintain their commitment because of other things that they like to do as well. I just feel that two sessions per week and the length of time that it runs for is possibly a bit too much to keep the attendance up.

School contact 72

Many dance instructors felt that two sessions per week was not typical for after-school clubs. One session per week was favoured for maintaining attendance. One school contact suggested that delivering the intervention in short ‘themed’ sections may be beneficial for encouraging attendance and return to sessions.

They do things better in bite size…you'd have almost been better off breaking it down to five week projects and a meeting at the beginning of each one so everybody knew where they were.

School contact 62

### Session content

Session content relates to themes concerned with the delivery of sessions, including variety in session content and group work.

#### Variety in session content

The BGDP was designed to incorporate numerous dance styles. Session variety, was seen to be important for maintaining interest. The majority of dance instructors gave girls a choice of dance styles, an approach which gained approval from the girls:

She [dance instructor] asked us what types of things we wanted to do. Some people said contemporary, some people said breakdancing, so that's what we did which was good.

Focus group 53

#### Group work

Generally, group work was viewed positively by instructors and girls. Dance instructors felt girls enjoyed group work and it encouraged them to take ownership of the project:

With tasks and things like that I kind of just gave them the choice in their groups so they just kind of got on with that.

Dance instructor 32

Girls found group work enjoyable and it appeared to help improve their dance and team working skills.

We like worked well in the group. There were like no arguments.

Focus group 53

Group work was seen to be beneficial to instructors and girls. Notably, it gave girls a sense of ownership over the project and developed their leadership skills. For dance instructors, it helped them manage the varied levels of competence within the group, and was perceived be a useful strategy for managing inconsistent attendance.

When it came to choreography and teaching other people that's when they took their ownership more so of the club.

Dance instructor 21 & 51

There was a tendency for instructors to allow participants to choose their own groups at the beginning of the project and then mix the groups once they felt comfortable with one another.

The first sessions I normally, if I'm doing group work, let them go with who they want to go [with] and then like when they feel more confident I kind of change it up a bit so they get to know new people.

Dance instructor 53

### Project organisation

Project organisation relates to open enrolment, parental involvement, facilities and communication and management arrangements.

#### Open enrolment

All participant groups suggested that an ‘open enrolment’ policy, allowing girls to ‘drop in’ to sessions anytime during the 20 weeks would be a good way to maintain attendance. Teachers stressed the importance of friends in ensuring continued attendance.

So we say ‘it's netball on Tuesday, anyone can come along. If you played for the primary school come along and see what it's like […] bring your friends’. If only three year sevens turn up we'll say ‘right, you're challenge is, next week you have to bring a partner’. And then when six turn up I say ‘right, you have to bring a friend’. So that's how we kind of do it. ‘Grab your friends, all come together’ because it's very much a friendship thing.

School contact 42

Open enrolment was viewed as a feasible strategy as long as the project was mindful of new people joining and causing disruption to the existing group (and its progress).

Perhaps you might say ‘you could join in after half term’ or ‘you can join in once we've finished this dance’. That's what I do at some schools.

Dance instructor 62

#### Parental involvement

School contacts suggested that increasing parental involvement in future after-school interventions may be beneficial. Generally it was recommended that increased parent awareness of the project may improve retention.

If you're going to roll it out, I think it has to be something a little bit more, towards the parents, like ‘you have to commit to it’. I think, yeah, that maybe just writing to the parents and when the kids stop coming sending a letter to the parents and saying ‘your child hasn't attended and I would really like them to come back’.

School contact 61

The advantage of increased parental involvement was outlined by some girls who described being encouraged to attend sessions by their parents.

Well when I said that I wanted to quit Active 7 she was like, ‘it is healthy for you and you should think about going again and don't stop it’.

Focus group 51

Similarly, dance instructors somewhat attributed attendance to parental encouragement and one instructor thought girls appeared to be motivated to attend because their parents told them to.

I think their parents kind of told them to be there.

Dance instructor 21 & 51

#### Facilities

Pupils found having the dance sessions on school premises convenient. The school teaching space was appropriate because they did not have to travel.

It was always in the same room. Like say if we had to change rooms every single time I think that would have been a bit harder but I like it how it was just in one room.

Focus group 32

In some instances there were problems with the facilities. These included the room temperature and ventilation, access to toilets and changing facilities, and in one school a teaching space that had a viewing gallery. Having to change venue due to conflicting activities (eg, examinations) was also inconvenient and gave dance instructors the impression that their session was not as valued by the school as they wished.

There's a bit at the top [of the dance studio] […] people used to stay here after school and they used to come in and like start watching […] So everyone would have stopped because they got embarrassed.

Focus group 42

[Having to move venue] was always really confusing because you'd sometimes lose some girls because they couldn't find you or you'd lose time faffing around trying to figure out what room you were in.

Dance instructor 23

#### Communication and management arrangements

The majority of dance instructors described a good working relationship with their school contact. School contacts were seen to be supportive of the instructor and the study. In some cases, school contacts observed dance sessions; this was viewed positively by dance instructors.

I emailed [the school contact] once about the level of noise the girls had, and then I saw him like a session or two later and he was like ‘do you want me to have a quick pop in?’ and I was like ‘yes, that would be great’. So he was really up for it.

Dance instructor 21

One school contact was keen to learn from the dance instructor's teaching practices.

I just go down a couple of Tuesdays and join in with [dance instructor] because she's quite a good teacher and it's always good to learn some new stuff.

School contact 32

Conversely, in two schools dance instructors did not feel adequately supported by their school contact. This was largely attributed to poor communication and lack of knowledge of the year group.

Often I'd like ask her to come in, especially at the beginning, I said “can you come and sit in the lessons?” and she wouldn't reply to my emails.

Dance instructor 21 & 52

She didn't know any of the Year Sevens so that meant it was quite difficult for her to communicate with them about sessions.

Dance instructor 53

## Discussion

This study elicited three key themes that affected delivery of the BGDP. The recruitment process, session content and intervention organisation were identified as specific areas where improvements could be made. Each of these themes and the potential implications/solutions for them are presented in table 2 and discussed below.

**Table 2:** Recommendations for future physical activity (PA) programmes delivered during the extracurricular period

Issue	Problem (or potential problem) encountered	Potential solution
Recruitment	*School contacts*: Many contacts were not familiar with the participants (as they had not taught them yet) which made data collection (particularly the return of accelerometers) difficult School contacts not communicating with dance instructors (over intervention issues) and the study team (over data collection)	To facilitate data collection, future recruitment of school contacts that are familiar with the participants (eg, Head of their year group) is recommended A calendar of tasks and requirements—with details on estimated time input—for school contacts may better prepare them for the role. A protected time allocation (weekly or monthly) for school contacts would ensure they can communicate with intervention deliverers and study staff, thus better equipping them for the time demands of the role and giving more time to resolve any problems
	*Dance instructors*: It was difficult to recruit appropriate intervention deliverers for the requirements of participants (may specialise in one form of dance, teach different age groups/genders/abilities etc) Intervention deliverers unable to deliver all intervention sessions	Endorsements from other dance instructors, schools, and dance agencies are useful for recruitment. Recruitment workshops, whereby the project can be introduced to dance instructors, are also recommended. Observation of intervention deliverers before recruitment is desirable but time and cost dependant Reserve deliverers should be recruited to cover absences and in the event of deliverers withdrawing from the study, these can be called on as replacements
	*Girls*: Confusion of receipt of voucher for participation in measurements with being paid to attend the intervention sessions. Friend involvement is an important factor influencing the recruitment of participants	Participants must be explicitly told (verbally and in writing) of the exact purpose of incentives to participate in data collection and what they will be received for Our results suggest that recruiting existing friendship groups and promoting the importance and esteem of the university-led research in the participants’ schools may help to achieve a greater buy-in from potential participants. Avoiding recruiting children in the first few weeks of term may be beneficial as they are likely to be more ‘settled’ into their friendship groups by this time
Timetabling	Clash of timing of school activities and intervention sessions Children require sufficient time to get changed and arrive punctually for the scheduled intervention start time	A calendar of after-school events, extracurricular activities, and the requirements of participants (including factoring in time to reach sessions from previous classes) should be sought to reduce overlap of activities. School contacts should be encouraged to avoid scheduling intervention sessions on days that other activities run (or are likely to run in future—based on previous years’ scheduling)
Session quantity	Two sessions per week was seen as too great a commitment for some participants. The total number of sessions (n=40) was also considered too many for some	The delivery of interventions in ‘blocks’ of sessions—covering different themes—should be considered ahead of future delivery The frequency of sessions and the overall number of sessions must be thoughtfully considered in light of the participants (age, existing ability and any other potentially important variables), achieving sufficient exposure to the intervention in order to achieve behaviour changes, and the timetable of schools
Session variety	Participants want to cover different material/activities. Activity choice should reflect participants’ desires while being achievable under the deliverer's skill set and capability	Offer participants genuine ‘choice’ over activities such as dance styles, and provide context-specific approaches to delivery, tailored to the needs and the requirements of the specific school
Group work	Group work is liked by participants	Embedding group work into interventions is likely to be helpful and may improve participants’ sense of ownership if they are able to select their own groups
Open enrolment	One phase of participant enrolment (prebaseline measurements) may unnaturally restrict participation	Open enrolment, whereby participants can ‘drop in’ to sessions anytime, rather than signing up to the intervention at the onset only, should be considered to mirror usual school provision. Allowing participants to join midway through the intervention period may improve retention, increase diversity, and give more people exposure to the intervention. In a trial setting this may be difficult logistically unless *all* potential participants take part in baseline measures
Parental involvement	Parents are an important influence over children and are likely to (or have the potential to) affect attendance	Developing strategies for parental support for extracurricular PA programmes should be incorporated into intervention design. Increased parental awareness of study aims and commitments may improve recruitment rates and attendance
Facilities	School-based interventions are limited by the facilities a school has	The ability to respond to participant desires regarding adaptable facilities (ie, heating, drinks provision, changing facilities) and act on them is encouraged in the future delivery of PA interventions. Choice over when windows/doors are opened, heating turned on, or whether a session is conducted outside (if feasible) should be discussed with participants School facilities are used for different purposes at different times of the year (ie, for school productions at Christmas and examinations in the summer). Attempts to protect the use of facilities for intervention sessions should be considered, but is likely to be difficult
Communication/management	Poor communication between any two stakeholders (study team, school contact and intervention deliverer) can have negative consequences for sessions	Recruiting school contacts who want to be involved rather than being pressurised may foster better communication (however, this would be difficult to achieve in reality, other than targeting relevant subject staff). Writing formal guidelines on regular updates between dance instructor and school contact/study team may resolve ongoing problems and/or re-engage children who have stopped attending. Any added burden on those delivering the intervention or school contacts should be given extensive consideration and avoided if possible

Different methods of recruitment were required for each stakeholder group. Familiarity with participants taking part was important among school contacts providing the link between schools, dance instructors and the research team. This suggestion is pertinent given the complexities many school contacts faced when ‘chasing’ research participants to encourage attendance (a task exacerbated by an unfamiliarity with the students). In future, it would be helpful to specify in detail what the role of school contact entails, highlighting the time needed for individual tasks and when they need to be completed (although over-burdening the contact with information should be treated with caution). Asking school contacts to allocate time for liaison with study staff/intervention deliverers may better prepare them for the role and improve delivery. For girls, targeting peer groups was considered sensible and a realistic method for attracting participants. Our findings also suggest that espousing the credentials of the project to instil a type of project ‘privilege’ may provide a further incentive for participation. This finding is consistent with previous research that suggests it is useful to identify and garner the support of influential ‘opinion makers’ to create a ‘buzz’ around the study.^24^ Such recruitment campaigns should be considered as part of the design of future after-school PA interventions.^24^ Assigning self-employed dance instructors to schools can be logistically difficult as many work on short-term contracts and continuously bid for work. This makes attending two sessions per week over a 20-week period a difficult commitment. Indeed, one instructor had to be replaced mid-way through the intervention. We would advise recruiting a bank of reserve instructors to ensure cover is always available.

School contacts selected the days and start/end times for intervention sessions. Dance instructors were assigned to schools to proximity and availability on session days. Subsequently, however, many schools had competing after-school activities on the same day as intervention sessions. Additionally, some children and dance instructors reported about sessions starting too soon after the school day ends. As such, greater consideration needs to be given to the scheduling of sessions, with the study manager and school contacting working through a set of potentialities to find a convenient and protected time.

A number of participants suggested that the intervention intensity, both in terms of the number of sessions per week and the duration of the intervention period, may have been too great a commitment to sustain attendance and was somewhat discordant with usual school provision. One solution suggested by a school contact, was to implement the project in 5 week modules where different dance styles are implemented in each block. As such, future projects may wish to employ structures that mimic usual school provision, and ensure intervention implementers and school staff deliver after-school interventions via this approach.

Open-enrolment was highlighted as an approach that may improve attendance and fluidity of delivery. However, it was noted that this would require dance instructors to carefully manage the dynamics of introducing new participants to the existing group, including the potential disruption this could cause. This suggestion is reasonable for mainstream delivery of the project, but the use of this strategy in a trial setting raises a problem in that participants receiving the intervention would change during the intervention period and, as such, intention-to-treat analyses would not be possible. This issue is therefore a reflection of broader debates in relation to the internal and external validity of public health interventions.^25–27^ Although measures that maintain the rigour of a trial, such as limiting recruitment numbers, may increase internal validity, it may limit the external validity. Hence, although restricting the number of participants to those who signed up at baseline was a necessity, it may not reflect usual practice, whereby children are able to attend or ‘drop-in’ to after-school clubs at times convenient to them. Further work examining the use of modified intervention design for real-world public health interventions may be warranted.^27–29^

Future delivery of after-school PA interventions may benefit from a greater awareness of existing school events. Study staff may wish to ask schools for the current and previous year's schedule of activities and check this against the planned intervention sessions, in the hope of identifying any current or future overlaps. While this will not stop all withdrawals, it may reduce instances of children signing-up when they are likely to drop out at a later date (thus leaving space for children who may follow the intervention through to the end). Identifying prospective timings convenient to girls is significant, given the multiple challenges already associated with implementing PA interventions during school hours.^7^ ^30^ ^31^

The call for greater variety (eg, a preference for differences in dance styles) in session content highlights the complexities of implementing interventions in distinct settings. Settings-based approaches to PA interventions have been highlighted elsewhere.^32^ ^33^ These findings support the need for a more ‘context based approach not only during data collection, but also for defining basic research constructs and questions’.^34^ Findings highlight the significance of ensuring variety in session content and for influencing participation and attendance across schools. Different dance styles appealed to different girls. While the programme set out to offer girls input into dance styles, music and pace of progression, the effectiveness of this approach relies on employing dance instructors who are willing and able to teach a range of dance styles. While this was largely the case in the BGDP, it is important that the recruitment of intervention deliverers ensures that their skills allow them to deliver the planned content *and* be flexible to input from the participant group. The group work component of the intervention was valued by participants and dance instructors as it fostered ownership of the project, helped the instructor cope with various levels of competence within the group, developed girls’ leadership skills and mitigated against inconsistent attendance. This finding is consistent with the broader literature associated with the principle of relatedness within Self Determination Theory.^35^

Parents were identified as an important source of support for behaviour change that was not utilised in this study. This finding is consistent with previous work which has identified parents as a potentially important feature of PA behaviour change.^36–41^ Parents represent a potential ‘lever’ that can be used to influence the PA levels of children, and as such work that specifically focuses on how to engage parents in providing positive support for extracurricular PA programmes is warranted.

### School culture impacts on the intervention delivery

Through our extensive engagement with school contacts, dance instructors, and girls, we observed (but did not formally assess) an implicit school ‘ethos’ or ‘culture’ which affected the intervention delivery and may have influenced the themes discussed above. The main school culture factors that appeared to affect the acceptability of the study were the school's organisational structure and communication between staff, the school's expectations of pupil behaviour and attendance, and the role of the school contact. When approaching schools to recruit participants, differences in attitudes were discernible from the outset, with some schools having a room booked and time set aside, and others forgetting the meeting had been arranged. Intervention logistics were also affected by distinct school cultures. Prior to recruitment, schools specified the days that intervention sessions would run so at the point of recruitment all girls knew the time and days on which they would receive dance sessions. In one case the school contact changed the days on which sessions ran. This school had the lowest average attendance, in part because many participants were not able to attend on the rescheduled day. Additionally, the same school contact set up a competing after-school club on the same day as the revised sessions. On paper, all schools encouraged consistent attendance, but in reality the expectations on girls varied widely between schools (Personal communication, S J Sebire, *et al*, 2016). Some school contacts expected girls to attend and were proactive in their approach in supporting them to do so. Others felt that their lack of familiarity with the girls made it difficult for them to encourage them, resulting in fewer, more ineffective attempts. All issues discussed above are reflective of the heterogeneity in the ethos of the participant schools. The findings highlight the fundamental importance of being aware of, and accounting for, the diversity of schools’ needs in planning after-school PA interventions.^42^

We encourage researchers to give greater consideration to the ‘school context’.^21^ Determining what contextual factors are important for a given study are difficult to establish preintervention and any formal assessment of the impact of school context will be difficult. Researchers should keep field notes of interactions with school and record issues that facilitate or hinder the study and intervention. Such a pool of knowledge from different studies and contexts may be the foundations on which more formal assessments of school context can in the future be made.

## Strengths and limitations

This study provides new information on factors which affect the delivery of after-school PA intervention. Although data used in this study are primarily focused on dance, we hope the findings will have future utility for researchers or practitioners operating within the broader field of PA interventions. A major strength of this research lies in the in-depth exploration of qualitative data obtained from a range of stakeholders. Data analysis was conducted by a team of researchers experienced in qualitative research. Two researchers participated only in the analysis stage of the process evaluation, and hence afforded a degree of objectivity, untainted by previous involvement in data collection. The total number of participants (n=78) is large, and there was evidence of data saturation. It should be noted that the findings represent issues associated with trial implementation, rather than the actual experiences of after-school PA interventions. Hence, they should not be considered a checklist for challenges associated with PA interventions. A limitation is that the issues that we report are grounded only in the experiences of stakeholders involved in one intervention, which was delivered to girls only in a relatively small area of the South West. As such, while many issues are applicable to the planning and implementation of broader after-school PA interventions it is possible that other interventions would reach different conclusions. We encourage other intervention planners and delivers to conduct detailed and reflective process evaluations and further contribute to the knowledge base for which school-based interventions can be improved.

## Conclusions

This study provides information on factors associated with BGDP delivery and identifies lessons which may be applied to future after-school PA interventions. Although after-school PA interventions hold promise in increasing PA levels among adolescent girls, there is a need to implement them in ways that are appropriate to the needs and requirements of schools and girls. Our findings suggest that implementation processes need to be contextually specific and the recommendations proposed in this study may have utility in achieving this objective.
